# A Novel Two-Step Approach for Retrieval of an Impacted Biliary Extraction Basket

**DOI:** 10.1155/2012/435050

**Published:** 2012-08-16

**Authors:** Calvin H. Y. Chan, Fergal Donnellan, Godfrey C. K. Chan, Michael F. Byrne

**Affiliations:** Division of Gastroenterology, Vancouver General Hospital, University of British Columbia, Vancouver, BC, Canada V5Z 1M9

## Abstract

Biliary extraction baskets are a commonly used instrument for the removal of choledocholithiasis in endoscopic retrograde cholangiopancreatography (ERCP). Impaction of the extraction basket is a recognized complication of ERCP, and is usually the result of discrepancy between the size of bile duct stone and the diameter of the distal bile duct. Whilst mechanical lithotriptors can be used to crush the stone or break the wires of the basket to allow its release, failure of the lithotriptor device can occur. We describe the case of a 59-year-old gentleman who had an ERCP performed for choledocholithiasis. Basket impaction was encountered, and the mechanical lithotriptor failed to dislodge the stone/basket complex. A two-step technique involving balloon dilatation and forceps manipulation of the basket was applied to successfully dislodge the impacted basket. We believe this simple and safe technique should be adopted to rescue impacted biliary extraction baskets to avoid the need for potential surgical removal.

## 1. Introduction

One of the most common indications for endoscopic retrograde cholangiopancreatography (ERCP) is choledocholithiasis. Stone extraction baskets are commonly used to remove biliary tract stones. One recognized complication of stone extraction is impaction of the extraction basket in the bile duct, a result of stone to distal duct size discrepancy. Whilst some baskets have a release mechanism to reduce the risks of impaction, other baskets rely on the use of an intra-or extraendoscopic mechanical lithotriptor to either crush the stone or break the wires of the basket to allow its release. The mechanical lithotriptor is often considered a final resort before surgical removal of the impacted basket and stone complex is considered necessary. We describe a case of an impacted extraction basket removed by a novel technique using step and balloon dilatation combined with rat-tooth forceps removal, after failure of deployment of a mechanical lithotriptor.

## 2. Case Report

**Figure 1:**
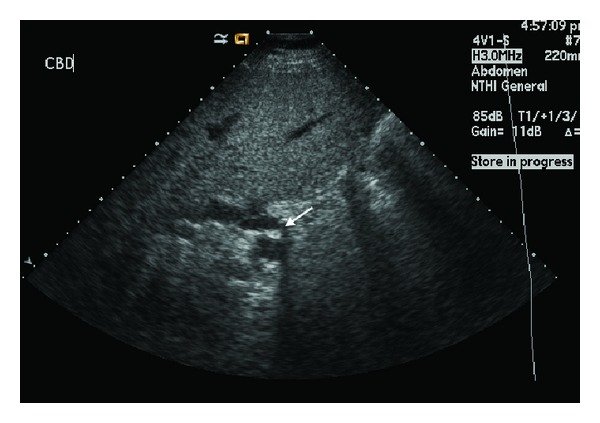
Transabdominal ultrasound of common bile duct (CBD) with CBD stone (arrow) and associated posterior acoustic shadowing.

A 59-year-old gentleman with no other significant background medical history presented to the emergency department of Vancouver General Hospital with a 12-hour history of painful jaundice. He was afebrile and there were no clinical features of sepsis upon presentation. Liver function tests demonstrated a bilirubin of 134 *μ*mol/L, GGT of 677 U/L, and ALT of 239 U/L. His inflammatory markers were normal. A transabdominal ultrasound demonstrated mild intrahepatic duct dilatation with a dilated common bile duct measuring 10 mm in diameter and evidence of a 10 mm stone in the mid CBD (Figure 1). The gallbladder was partially distended with no wall thickening or pericholecystic fluid and indefinite for cholecystolithiasis.

**Figure 2:**
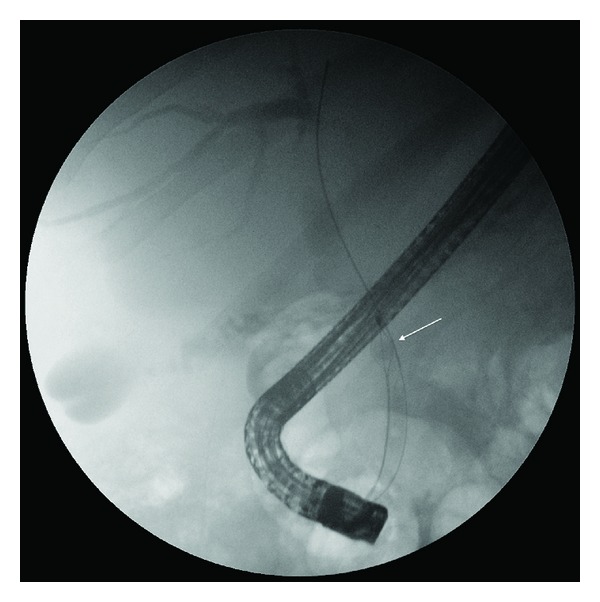
ERCP demonstrating the biliary extraction basket around a stone (arrow) within the CBD.

A diagnosis of obstructive jaundice secondary to choledocholithiasis was made and the patient proceeded to an ERCP with planned removal of the sonographically visualized stone. Biliary cannulation was difficult. A needle knife fistulotomy was required to facilitate biliary access, and subsequent cholangiogram demonstrated a 10 mm bile duct with an ill-defined 8 mm filling defect in the mid duct. The fistulotomy orifice was extended with a sphincterotome, and a 25 mm extraction basket (The Web extraction basket, Cook Inc, Winston-Salem, NC, USA) was used to extract the stone. Surprisingly, despite the apparent size compatibility of the stone with distal duct and sphincterotomised orifice (Figure 2), the basket became impacted within the intraduodenal portion of the bile duct.

**Figure 3:**
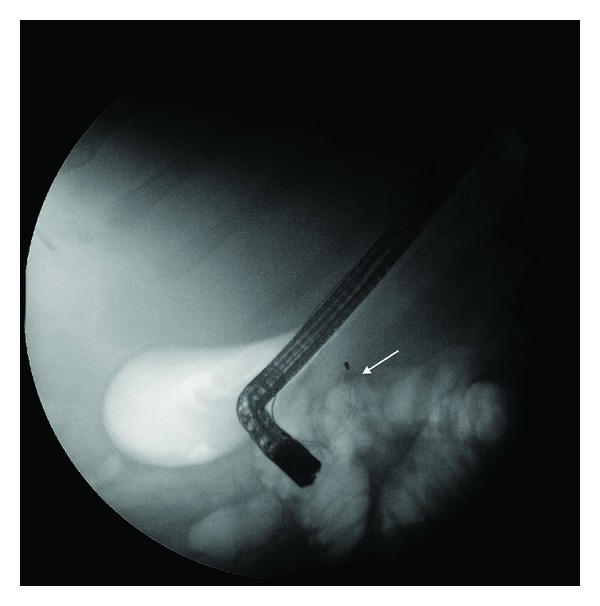
Basket/stone complex (arrow) lodged in the distal CBD after attempted mechanical lithotripsy.

The plastic sheath covering the basket wire was cut exposing the free wires of the basket, and a mechanical extraendoscopic lithotriptor was used (Soehendra lithotriptor, Cook Inc, Winston-Salem, NC, USA). Upon cranking the lithotriptor, the wires fractured at the handle, outside the oral cavity. The duodenoscope (JF 180, Olympus Optical Co Ltd, Toyo, Japan) was then reinserted into the duodenum and biliary cannulation was reattempted around the basket (Figure 3). However, due to the local edema, no instruments beside a guidewire were able to be advanced beyond the stone/basket complex. A second attempt at mechanical lithotripsy was performed with the use of a shorter Soehendra mechanical lithotriptor metallic sheath, but again, the wire fractured a second time at the handle. A decision was made to reattempt ERCP 48 hours later to allow the local edema to settle. The procedure was abandoned, the basket wire secured with dressing forceps and surgical tape outside the oral cavity, and the patient placed on prophylactic antibiotics.

**Figure 4:**
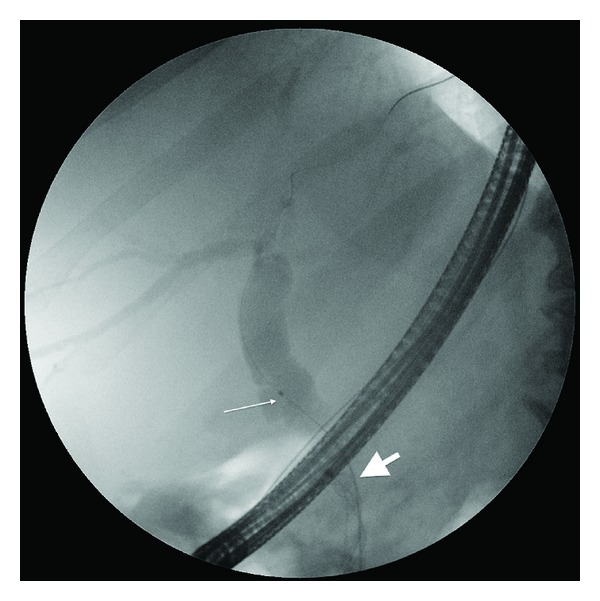
Insertion of a wire guided 8.5 Fr dilating catheter (thin arrow) beside the basket/stone complex (thick arrow) in the CBD.

**Figure 5:**
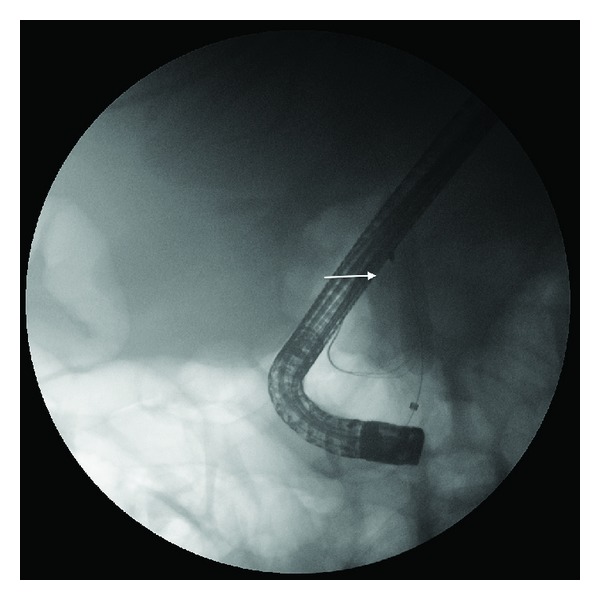
Inflation of a 12 mm dilating balloon beside the stone/basket complex (arrow) in the distal CBD.

**Figure 6:**
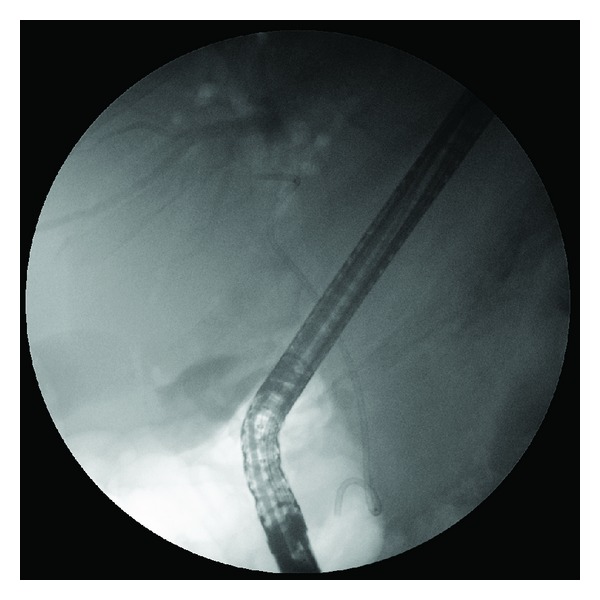
Double pigtail biliary stent within the CBD after basket removal.

The patient remained stable and ERCP was reattempted. Cannulation was reattempted with a sphincterotome (Dreamtome, Boston Scientific, Natick, Mass, USA), but once again only the guidewire was able to be advanced beyond the basket/stone complex. An 8.5 Fr biliary dilating catheter (Fusion Biliary Dilation Catheter, Wilson-Cook, Winston-Salem, NC, USA) was able to be introduced over the guidewire (Figure 4). This allowed a 12 mm dilating balloon (CRE Balloon, Boston Scientific, Natick, MA, USA) to be deployed at the level of the impacted basket (Figure 5). We then used rat-tooth forceps (Olympus Tokyo, Japan) to grasp the struts of the basket. The basket was easily dislodged. A 7 Fr 7 cm double pigtail stent was subsequently inserted as a precautionary measure against ductal trauma (Figure 6) and the patient was discharged the next day. He returned one month later with an uncomplicated clearance of his bile duct with an extraction balloon and removal of the stent.

## 3. Discussion

Biliary extraction baskets are made from metal wires and are available in a variety of sizes and configurations. Basket impaction is an uncommon but recognized complication of ERCP stone extraction, and represents a medical emergency. Whilst some baskets have a built-in safety mechanism to break upon excess force of closure, most baskets require an external crushing sheath such as the Soehendra lithotriptor to either crush the stone or break the basket wire to retrieve the device. Both external and “through the scope” lithotriptors are available [1, 2]. To avoid surgical removal, alternative techniques described include awaiting for spontaneous passage [3], the use of extracorporeal shockwave lithotripsy [4–6], intracorporeal electrohydraulic lithotriptor [7], extending the sphincterotomy with a needle knife [8], and tipping the basket tip using a second basket [9].

The use of rat-tooth forceps to maneuver the basket struts to slide beside the stone has been described [10], but this alone may be ineffective in a less-distended duct with a heavily impacted basket. Just as balloon sphincteroplasty has aided removal of large biliary stones [11], we describe a technique here combining two-proven modalities to safely remove an impacted basket. The purpose of the graduated dilating catheter was to allow a dilating balloon to be advanced beside the stone/basket complex. By dilating the sphincter and the distal duct, this allows the basket struts to be easily maneuvered by the rat-tooth forceps to slide beside the stone. The remaining basket should subsequently have adequate space to be retrieved. This simple combination of steps should be considered in patients with impacted baskets before consideration of surgical removal.
